# Divergent N Deficiency-Dependent Senescence and Transcriptome Response in Developmentally Old and Young *Brassica napus* Leaves

**DOI:** 10.3389/fpls.2018.00048

**Published:** 2018-02-01

**Authors:** Vajiheh Safavi-Rizi, Jürgen Franzaring, Andreas Fangmeier, Reinhard Kunze

**Affiliations:** ^1^Institute of Biology, Dahlem Centre of Plant Sciences, Free University Berlin, Berlin, Germany; ^2^Institute of Landscape and Plant Ecology, University of Hohenheim, Stuttgart, Germany

**Keywords:** autophagy, *Brassica napus*, leaf senescence, N remobilization, N-deficiency, oilseed rape, transcriptome, transcription factor

## Abstract

In the spring oilseed rape (OSR) cultivar ‘Mozart’ grown under optimal N supply (N_O_) or mild N deficiency (N_L_) the transcriptome changes associated with progressing age until early senescence in developmentally old lower canopy leaves (leaf #4) and younger higher canopy leaves (leaf #8) were investigated. Twelve weeks old N_O_ and N_L_ plants appeared phenotypically and transcriptomically identical, but thereafter distinct nutrition-dependent differences in gene expression patterns in lower and upper canopy leaves emerged. In N_O_ leaves #4 of 14-week-old compared to 13-week-old plants, ∼600 genes were up- or downregulated, whereas in N_L_ leaves #4 ∼3000 genes were up- or downregulated. In contrast, in 15-week-old compared to 13-week-old upper canopy leaves #8 more genes were up- or downregulated in optimally N-supplied plants (∼2000 genes) than in N-depleted plants (∼750 genes). This opposing effect of N depletion on gene regulation was even more prominent among photosynthesis-related genes (PSGs). Between week 13 and 14 in leaves #4, 99 of 110 PSGs were downregulated in N_L_ plants, but none in N_O_ plants. In contrast, from weeks 13 to 16 in leaves #8 of N_L_ plants only 11 PSGs were downregulated in comparison to 66 PSGs in N_O_ plants. Different effects of N depletion in lower versus upper canopy leaves were also apparent in upregulation of autophagy genes and NAC transcription factors. More than half of the regulated NAC and WRKY transcription factor, autophagy and protease genes were specifically regulated in N_L_ leaves #4 or N_O_ leaves #8 and thus may contribute to differences in senescence and nutrient mobilization in these leaves. We suggest that in N-deficient plants the upper leaves retain their N resources longer than in amply fertilized plants and remobilize them only after shedding of the lower leaves.

## Introduction

In the past three decades the worldwide oilseed rape acreage has expanded nearly threefold to 36 million ha and the production has increased even fivefold to 73 million tons in 2013 (Food and Agriculture Organization of the United Nations)^1^. In winter oilseed rape production fertilization with up to 200 kg nitrogen (N) ha^-1^ year^-1^ is common practice. Although oilseed rape (OSR) has a high uptake capacity for inorganic N, its nitrogen use efficiency (NUE; for definitions see Masclaux-Daubresse et al., 2010; Xu et al., 2012) is low. Only 50–60% of the applied N is recovered in the plants and at the time of harvest 80% of the total plant N is localized in the seeds (Schjoerring et al., 1995; Jensen et al., 1997; Malagoli et al., 2005a; Rathke et al., 2006). Accordingly, winter OSR production has a high N balance surplus that often exceeds the limit of 60 kg ha^-1^ year^-1^ that is effective since 2009 in Germany (Düngeverordnung)^2^ and the European Union (Nitrates Directive^3^). To meet these requirements without compromising seed yield, the development of cultivars with improved NUE at reduced fertilizer input is an important agricultural goal in OSR breeding.

Two factors determining the NUE are the N-uptake ability of the plants and the N-remobilization efficiency from old, senescing leaves during pod development and seed ripening. N-uptake increases in young plants approximately until flowering, but stagnates or even decreases during pod ripening and contributes only a minor fraction of the N in the seeds (Schjoerring et al., 1995; Rossato et al., 2001; Malagoli et al., 2005a; Gombert et al., 2010). Indeed, the majority of N required for seed filling and pod ripening is mobilized from senescing leaves and stems (Malagoli et al., 2005a; Gombert et al., 2010). Although the N-efficiency of winter OSR can also be enhanced by breeding cultivars with enhanced N-uptake ability (Schulte auf’m Erley et al., 2007), strengthening the N remobilization activity of leaves during the vegetative phase is a promising approach for improving the NUE of oilseed rape (Gironde et al., 2015). In a simulation model of N partitioning, Malagoli et al. (2005b) came to the conclusion that by optimizing N remobilization from leaves at lower nodes and N retranslocation from vegetative to reproductive tissues, OSR yield could be increased by 15%.

Yet, the shed leaves from lower nodes still have a high N content of up to 3.5% whereas leaves from upper nodes contain at the time of abscission only 1% residual N (Malagoli et al., 2005a). What limits N remobilization from early senescing leaves? Phloem loading of amino acids from degraded leaf proteins appears not to be the limiting step (Tilsner et al., 2005). In many winter OSR cultivars the onset of senescence and abscission of lower node leaves occurs already during the vegetative stages before the development of pods and seeds. This lack of sink organs supposedly leads to a low N remobilization rate from early leaves (Schjoerring et al., 1995; Rossato et al., 2001; Noquet et al., 2004; Malagoli et al., 2005a). Accordingly, winter cultivars with a delayed leaf senescence phenotype (‘functional stay-green’; reviewed in Thomas and Ougham, 2014) tend to have a higher N-efficiency (Schulte auf’m Erley et al., 2007; Gregersen et al., 2013; Koeslin-Findeklee et al., 2015a,b).

The onset of leaf senescence is regulated by multiple, endogenous and environmental factors, among them N deficiency (Gregory, 1937; Mei and Thimann, 1984; Masclaux-Daubresse et al., 2007; Bieker and Zentgraf, 2013; Koeslin-Findeklee et al., 2015b). However, the developmental response to N deficiency and timing of senescence initiation are not uniform throughout the plant body. During development of winter OSR plants senescence progresses sequentially from the bottom toward the top and the sink leaves in young plants later turn into source leaves during pod ripening (reviewed by Avice and Etienne, 2014). N-deprivation triggers earlier onset of senescence in older leaves, whereas in young leaves at higher nodes senescence is delayed (Etienne et al., 2007; Desclos et al., 2008). Thus, the spatially and temporally concerted modulation of senescence initiation is a promising target to improve the N-efficiency of OSR, but it requires a deeper understanding of the metabolic and transcriptional changes associated with leaf senescence initiation and progression in different parts of the plant. Koeslin-Findeklee et al. (2015b) identified in a study of transcriptomic changes following senescence induction by N-depletion in leaves from a lower node of two *B. napus* winter cultivars differing in their stay-green properties and N-efficiency, a large number of cultivar-specifically regulated, senescence-associated genes, but they did not address leaf-rank specific expression differences.

In this study we report that in the doubled haploid OSR spring cultivar ‘Mozart’ senescence progression and the effect of N-limitation are similar as in winter OSR cultivars and we present a genome-wide developmental transcription analysis of plants grown under standard or reduced N-supply. The developmental transcription changes in lower and upper canopy leaves of plants grown under low N-fertilization indicated that in old (source) leaves senescence was initiated earlier and this onset was accompanied by extensive transcriptional reprogramming. In contrast, in young (sink) leaves at a node below the inflorescence, transcriptional reprogramming was delayed in N-depleted plants. We identified transcription regulator, autophagy and protease genes that were specifically regulated in N-depleted lower canopy leaves or in upper leaves under ample N supply, and genes that were expressed senescence-associated in oilseed rape, but not in *Arabidopsis*. We hypothesize that some of these genes may have OSR-specific functions in N-remobilization during N-deficiency induced leaf senescence and contribute to differences in senescence execution and nutrient mobilization in upper and lower canopy leaves.

## Materials and Methods

### Plant Material and Growth Conditions

Oilseed rape spring cultivar *Brassica napus* cv. ‘Mozart’ plants (BSA Nr. RAS 502, supplied by Norddeutsche Pflanzenzucht Hans-Georg Lembke KG – NPZ, Hohenlieth, Germany) were cultivated in solid medium in growth chambers that simulated the daylight length and average daily temperature profile between 1991 and 2005 in South–West Germany from March 15th (day 0: sowing) onward (Supplementary Table 1). Light intensity (photon flux density) during daylight phases was approximately 1000 μmol m^-2^ s^-1^. The average CO_2_ concentration during illumination was 396 ppm which approximates ambient atmospheric conditions. During the dark phase the CO_2_ concentration increased by approximately 100 ppm. A more detailed description of nursing, growth and physiological parameters of the plants analyzed in this study are presented in Franzaring et al. (2011). Leaf disks from early developing leaf #4 (at 78, 85, 92, and 99 days after sowing, DAS) and leaf #8 (at 92 and 106 DAS) were collected from plants grown at optimal (N_O_) or low (N_L_) N supply. For optimal N nutrition, NH_4_NO_3_ was supplied in three equal gifts to each pot at germination (0 DAS; extended BBCH-scale stage GS0; Meier, 2001), 72 DAS (GS35) and 79 DAS (GS59) at an equivalent of 150 kg N ha^-1^ t. For N_L_ plants fertilizer gifts were reduced by half (75 kg N ha^-1^ t). For each leaf sample three biological replicates from different plants were collected. Before harvesting, relative chlorophyll levels of the leaves were determined using a Konica Minolta SPAD-502 chlorophyll meter. For each leaf, SPAD values from two positions were measured and averaged.

### RNA Isolation

After freezing and grinding the samples in liquid nitrogen, total RNA was isolated by a hot phenol method as described (Drechsler et al., 2015). Total RNA was purified further using the RNeasy Mini Kit (Qiagen, Hilden, Germany). RNA quality was monitored on an Agilent 2100 Bioanalyzer (Agilent Technologies, Santa Clara, CA, United States).

### *Brassica napus* Custom Microarray Design and Functional Annotation

The *Brassica napus* custom microarray was designed and processed as described in Koeslin-Findeklee et al. (2015b). Briefly, after a probe-preselection strategy established by ImaGenes GmbH (Berlin, Germany; now Source BioScience)^4^ (Weltmeier et al., 2011) 60,955 probes representing 59,577 targets (EST clusters termed in this paper *B. napus* ‘unigenes’) were selected for the production of microarrays in the Agilent 8 × 60k format. Microarray design (GPL19044) and expression data (Series entry GSE97653) are deposited in the NCBI Gene Expression Omnibus (GEO) repository. To assign putative functions to the 59,577 *B. napus* ‘unigenes,’ they were locally BLASTed (RRID:SCR_004870) against the TAIR10 *Arabidopsis thaliana* cDNA collection (Lamesch et al., 2012) using the BioEdit alignment editor^5^ (RRID:SCR_007361). Putative functions were attributed to *B. napus* unigenes based on the annotation of the most homologous *Arabidopsis thaliana* genes with a BLAST *E*-value ≤ 10^-6^. When multiple *B. napus* unigenes had the same *Arabidopsis* homolog, the unigene with the lowest *E*-value that is significantly regulated in any one sample was selected for further analysis.

### Processing and Bioinformatic Analysis of Microarray Data

Microarray expression data readouts were generated by the Agilent Feature Extraction software. The raw data files were processed, normalized and analyzed with the Bioconductor package LIMMA^6^ (RRID:SCR_006442; Smyth, 2004). The *read.maimages* function was used to load the data into an RGList object. Background subtraction and quantile normalization was performed followed by statistical analysis (*moderated t-test*). The average of replicated spots was calculated using the *avereps* function. A design matrix was built for the *linear modeling* function and the intensity values were applied as *lmFit* function. Contrast matrices representing comparisons between different harvest time points and N treatments were created and applied to modeled data for computing the statistical significance. Regulated unigenes (≥3-fold expression change and Benjamini-Hochberg-corrected *p*-value *P*_adj_ < 0.05) were clustered by their temporal expression profiles with the Short Time-series Expression Miner (STEM) software, RRID:SCR_005016, using default settings (Ernst and Bar-Joseph, 2006). Grouping of unigenes into functional categories was performed with the BAR Classification SuperViewer Tool w/Bootstrap^7^ (RRID:SCR_006748) using MapMan (RRID:SCR_003543) categories as annotation source (Provart and Zhu, 2003; Provart et al., 2003). Enriched GO-terms were identified with the DAVID Bioinformatics Resources 6.8 in the GOTERM_BP_DIRECT term compilation using default settings^8^ (RRID:SCR_003033; Huang et al., 2009a,b). Heat maps of differentially regulated genes were created using MultiExperiment Viewer (MeV)^9^ (RRID:SCR_001915; Saeed et al., 2006). *Arabidopsis thaliana* transcription factors were compiled from the AGRIS AtTFDB^10^ (RRID:SCR_006928; Yilmaz et al., 2011), autophagy-related genes from the Autophagy database^11^ (RRID:SCR_002671; Homma et al., 2011), and peptidases from the MEROPS database^12^(RRID:SCR_002671; Rawlings et al., 2016).

### qPCR Primer Design and Assay for *B. napus* ‘Unigenes’

Primers for quantitative real-time PCR (qPCR) were calculated by QuantPrime^13^ (Arvidsson et al., 2008) after importing the *B. napus* unigene assemblies (Supplementary Table 13). One μg DNase I-digested total RNA was used for cDNA synthesis using SuperScript III Reverse Transcriptase (ThermoFisher Scientific). qPCR reactions were performed in 5 μl total volume including 2.5 μl Power SYBR Green Master Mix (ThermoFisher Scientific), 0.5 μM forward and reverse primers and 0.5 μl cDNA. *UP1* and *UBC9* were used as reference genes (Chen et al., 2010). The thermal profile used for all qPCRs was: 2 min 50°C > 10 min 95°C > (15 s 95°C > 1 min 60°C)_40*x*_. Data were analyzed by the 2^-ΔΔ*Ct*^ method (Schmittgen and Livak, 2008).

## Results

### The Transcriptome Response to Reduced N Supply Differs in Early and Late Oilseed Rape Leaves

**FIGURE 1:**
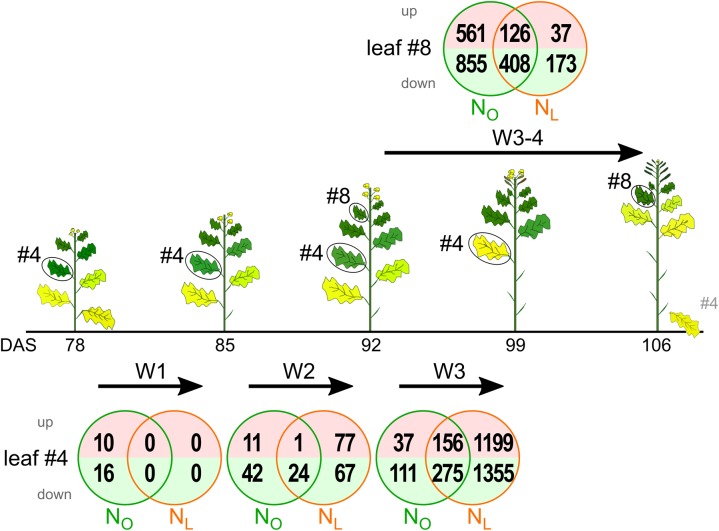
Transcriptome changes in oilseed rape leaf 4 (#4) and leaf 8 (#8) during development under optimal N supply (N_O_) and low N supply (N_L_). Leaves #4 and #8 were harvested between 78 and 106 days after sowing (DAS) as indicated below the sketches of the oilseed rape plants. The Venn diagrams display the numbers of upregulated and downregulated genes highlighted in light red and lime green, respectively. The examined developmental intervals are termed week 1 (W1), week 2 (W2), week 3 (W3) and weeks 3 + 4 (W3–4). Depicted are genes with significant, ≥3-fold expression changes (*P*_adj_ < 0.05, *n* = 3).

The aim of this study was to investigate if in spring oilseed rape (OSR) a mild N deficiency can be detected at the transcriptomic level, if the transcriptome response differs in developmentally older (source) leaves at a lower node and younger (sink) leaves at a higher node, and if the developmental response to N-deficiency resembles that in winter OSR cultivars. *Brassica napus* cv. ‘Mozart’ plants were raised under controlled conditions in a growth chamber under optimal N supply (N_O_) or N supply reduced by 50% (N_L_). Morphological, physiological and performance data of the same plants we investigated in this study were previously reported by Franzaring et al. (2011). The N_L_ conditions caused only subtle developmental and growth phenotypes (Supplementary Figure 1), flowering started on average only 2 days later than in N_O_ plants (Figure 3 in Franzaring et al., 2011), but seed yield was reduced (Table 1 in Franzaring et al., 2011) The early developing leaf #4 and the late developing leaf #8, located at the base of a flower developing side shoot, were harvested as representatives of old (source) leaves and young (sink) leaves, respectively. Leaf #4 was harvested at four different time points during development (78, 85, 92, and 99 days after sowing, DAS). Leaf #8 was harvested at the two time points 92 DAS and 106 DAS. Under both N treatments, at 92 DAS leaves #4 were still alive and attached to the stem, whereas at 106 DAS on most plants they were dead and shed (**Figure 1** and Supplementary Figure 1).

Transcription analysis was performed using a *B. napus* custom microarray representing 59,577 ‘unigenes’ (Koeslin-Findeklee et al., 2015b). For 54,095 (91%) of the *B. napus* ‘unigenes’ 19,185 homologs were identified in *Arabidopsis thaliana* (Supplementary Table 2). For only 5,522 of these *Arabidopsis* genes one single *B. napus* unigene is represented on the microarray, whereas for 71% more than one *B. napus* unigene exist (Supplementary Table 3). These unigenes include representatives in each of the 66 biological categories in the MapMan metabolic pathway visualizer (Thimm et al., 2004). In 41 (sub-)categories more than 70% of the corresponding genes are represented by a homologous *B. napus* unigene (Supplementary Table 4).

Under optimal N supply in leaf #4 the number of *B. napus* unigenes up- or downregulated relative to the previous harvest time point with significant (*P*_adj_ < 0.05) and ≥3-fold expression changes progressively increased from week 1 (26 genes) to week 2 (78 genes) to week 3 (579 genes) of the observation period (**Figure 1** and Supplementary Table 5). The same trend, but with a much steeper increment, was observed in plants that were grown under reduced N fertilization. In the first week the transcriptome did not change at all, whereas 1 week later 169 regulated genes appeared and in week 3 the number of regulated genes jumped to 2,985. In both growth conditions and all time intervals the downregulated genes outnumbered the upregulated genes.

In the upper canopy leaf #8, 1,950 genes were up- or downregulated in N_O_ plants and 744 in N_L_ plants. Thus, the relation of regulated genes in N_O_ and N_L_ plants was inverse compared to leaf #4. Also in leaf #8 the downregulated genes outnumbered the upregulated genes under both N fertilization regimes.

### Reduced N Supply Correlates with Differential Senescence Progression in Lower and Upper Canopy Leaves

The observed massive increase in gene regulation might indicate the onset of leaf senescence, which is known to be accompanied by transcriptome reorganization (Buchanan-Wollaston et al., 2005; van der Graaff et al., 2006; Koeslin-Findeklee et al., 2015b). We therefore tracked senescence initiation by measuring chlorophyll content and expression of chlorophyll A/B binding protein gene *BnCAB1*, *Brassica napus* drought 22 kD protein gene *BnD22* and the senescence associated genes *BnSAG12-1* and *BnSAG2* by qPCR.

**FIGURE 2:**
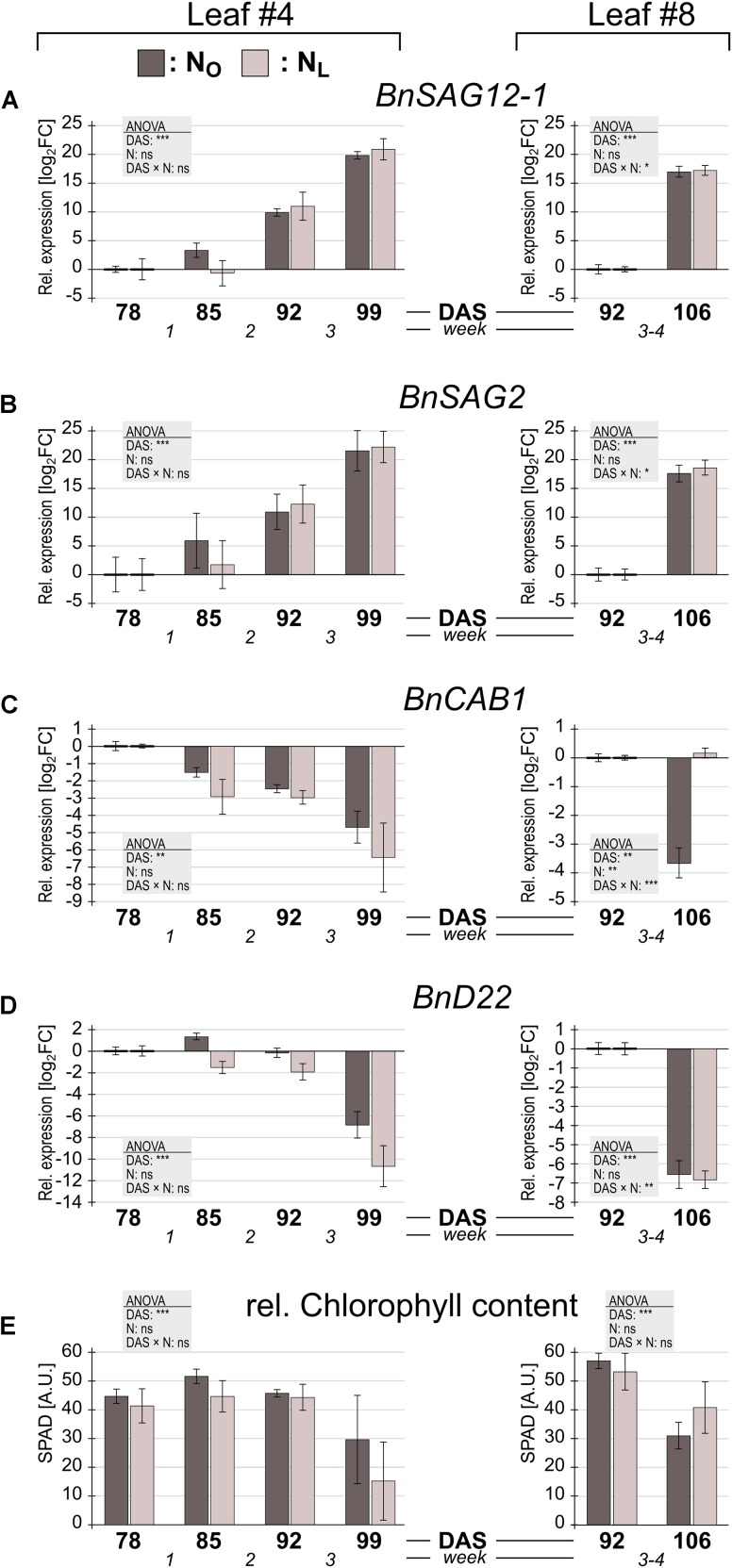
Senescence marker gene expression and chlorophyll content in *Brassica napus* leaves of plants grown under optimal or reduced N supply. *B. napus* leaves #4 and #8 of plants grown under optimal N supply (N_O_; dark gray columns) or under reduced N supply (N_L_; light gray columns) were harvested at the indicated days after sowing (DAS). Relative expression levels were determined by qPCR. For leaf #4, expression changes relative to the level at 78 DAS, and for leaf #8, expression changes relative to the level at 92 DAS are shown of **(A)** senescence associated gene *BnSAG12-1*, **(B)** senescence associated gene *BnSAG2*, **(C)** chlorophyll a/b binding protein gene *BnCAB1* and **(D)** *B. napus* gene *BnD22*. **(E)** Relative chlorophyll contents at each harvest time point are shown as SPAD values. Error bars indicate the standard error of the means (*n* = 3). Significant expression differences by N treatment, over time and by interaction of the two parameters was calculated by two way ANOVA (^∗^*P* < 0.05; ^∗∗^*P* < 0.01; ^∗∗∗^*P* < 0.001). Subsequently a Tukey’s HSD *post hoc* test was done to identify significant differences between all different harvest time points (Supplementary Table 6).

In leaves #4 of N_O_ plants, *BnSAG12-1* (**Figure 2A**) and *BnSAG2* (**Figure 2B**) expression by trend increased already in week 1 and continued to increase throughout weeks 2 and 3. In N_L_ leaves, upregulation of these two genes started only in week 2. Under both N treatments *BnCAB1* transcription appeared to decline in week 1 (**Figure 2C**), but the expression change was not significant (Supplementary Table 6). *BnD22*, whose expression level was approximately 3.5-fold higher under low N-conditions at the beginning of the observation period than under optimal N supply (Supplementary Table 5) as has also been reported by Desclos et al. (2008), displayed no significant expression change in weeks 1 and 2 (Supplementary Table 6), but a rapid decline in week 3 (**Figure 2D**). Expression of both *SAG*s and *CAB*, but not *BnD22*, indicated upcoming senescence one to 2 weeks before also a decline in chlorophyll was measurable (**Figure 2E**).

In leaf #8, the equal upregulation of *BnSAG12-1* and *BnSAG2* and downregulation of *BnD22* in N_O_ and in N_L_ leaves indicates that senescence has started during weeks 3–4. The regulation of *BnCAB1* was strikingly different in N_O_ and N_L_ leaves #8. Under low N supply *BnCAB1* expression was maintained, whereas under optimal N supply its transcription declined.

Neither in the lower or the upper leaves the differences between N_L_ and N_O_ plants in senescence marker gene expression and chlorophyll content were significant, whereas the expression pattern of *BnCAB1* is very different in N_L_ and N_O_ leaves #8. We therefore compared the expression levels of the 110 OSR homologs of *A. thaliana* photosynthesis-related genes (PSG) on the microarray (Supplementary Table 7). In the optimally N-supplied leaves #4, none of these genes were significantly up- or downregulated between 78 DAS and 99 DAS (except one downregulated gene in week 2). In striking contrast, in leaves #4 grown under reduced N fertilization, 94% of the PSGs were downregulated (4 PSGs in week 2 and another 99 PSGs in week 3). In the upper canopy leaves #8 the pattern was opposite: in N_O_ leaves 66 PSGs were downregulated compared to 11 downregulated PSGs in N_L_ leaves.

In summary, these data and the higher total number of up- and downregulated genes in N_L_ leaves #4 (**Figure 1**) indicate that a mild N deficiency leads not to a significant earlier initiation, but to a more rapid progression of senescence once it has started. In contrast, in the upper canopy leaf #8 mild N deficiency causes a delay in senescence progression. Thus, in spring OSR the effect of N-deprivation on senescence in older and younger leaves is similar as in winter OSR (Etienne et al., 2007; Desclos et al., 2008).

### N Fertilization-Dependent Gene Expression

**FIGURE 3:**
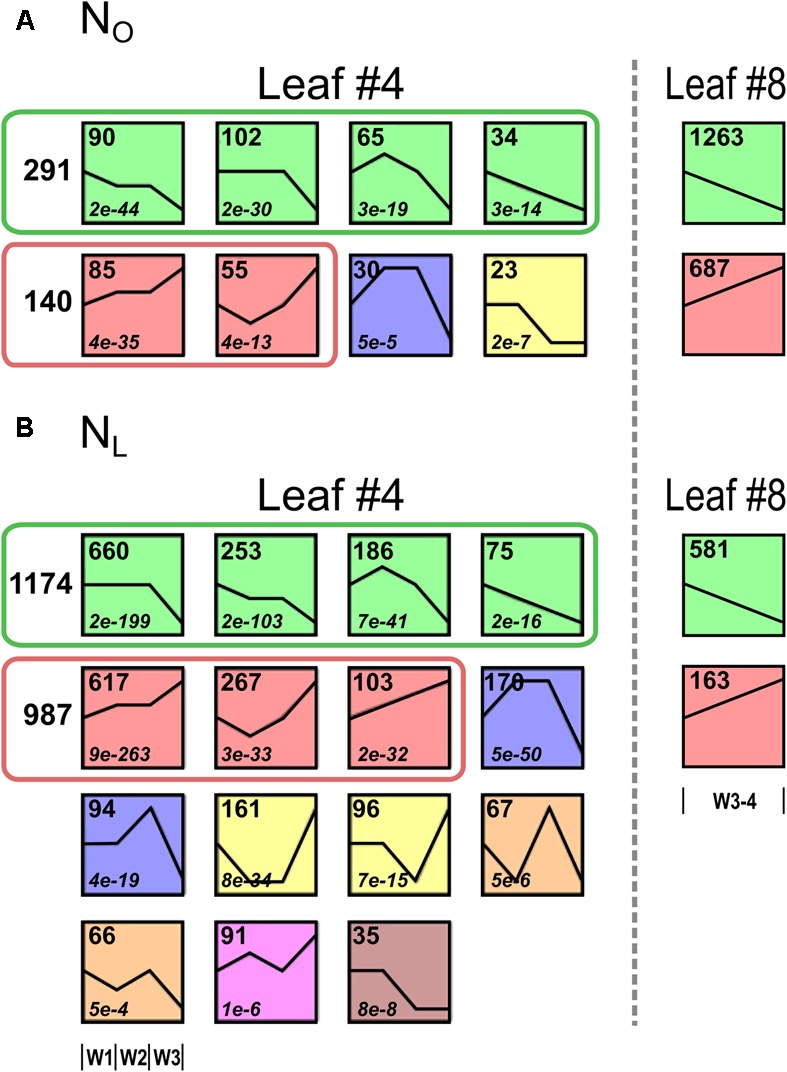
Clustering of regulated genes with similar temporal expression profiles in leaf #4. The leaf #4 genes regulated during development under optimal and reduced N supply were clustered according to their temporal expression profiles. The mean expression values (log2) of all differentially regulated genes (≥3-fold expression change in W1, W2, or W3 and *P*_adj_ < 0.05) were grouped by STEM using default settings. Each box represents one of 50 predefined expression profiles. Depicted are only profiles with a statistically significant number of genes assigned. Profiles with the same color are similar and defined as one cluster. The number of genes belonging to each model expression profile is shown in the top left corner of each box. **(A)** Of the 655 regulated genes under optimal N supply (N_O_), 484 genes are allocated to four clusters. Two hundred and ninety one genes are allocated to one ‘downregulated’ cluster that is subdivided in four profiles (green boxes) and 140 genes are allocated to one ‘upregulated’ cluster subdivided in two profiles (red boxes). To the right the numbers of up- and downregulated N_O_ leaf #8 genes are shown. **(B)** Of the 3111 regulated genes under reduced N supply (N_L_), 2771 genes are allocated to seven clusters. 1174 genes are allocated to one ‘downregulated’ cluster that is subdivided in four profiles (green boxes) and 987 genes are allocated to one ‘upregulated’ cluster subdivided in three profiles (red boxes). To the right the numbers of up- and downregulated N_O_ leaf #8 genes are shown.

To identify genes with similar expression change profiles during development under optimal and reduced N supply in the lower canopy leaf #4, the regulated genes were clustered by their expression profiles and assigned to 50 predefined model temporal expression profiles (Supplementary Table 8). Of the 665 genes up- or downregulated in N_O_ leaves #4, four clusters with eight profiles had a statistically significant number of genes assigned (**Figure 3A**). Overall, 291 of the genes (44%) are allocated to a cluster of four downregulated profiles and 140 genes (21%) fall into a cluster of two upregulated profiles. In leaves #4 of N_L_ plants, 2941 genes (94% of the 3111 regulated genes) are assigned to 15 model temporal expression profiles with a statistically significant number of genes (**Figure 3B**). Of these, 1174 genes (38%) are allotted to a cluster of four downregulated profiles and 987 genes (32%) to a cluster of three upregulated profiles, respectively. A conspicuous contrast between the transcriptomes of the developmentally younger upper canopy leaves #8 compared to the older leaves #4 is that in leaves #8 a higher number of genes is regulated during the 2 weeks observation interval in plants grown under optimal than under reduced N supply (**Figure 1**).

### Functional Classification of N Fertilization-Dependently Regulated Genes

**FIGURE 4:**
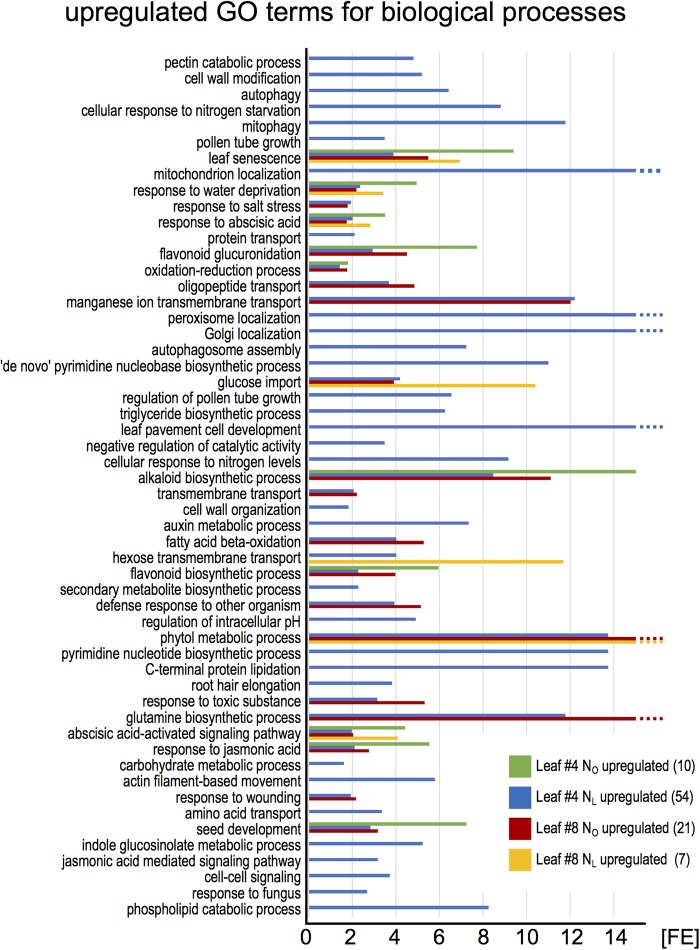
Enriched Gene Ontology terms among upregulated leaf #4 genes. The upregulated genes in all samples (red pictograms in **Figure 2**) were analyzed for enriched Gene Ontology terms using the DAVID Bioinformatics Resources 6.8. The blue bars represent all significantly enriched GO_BP_DIRECT terms in N_L_ leaves #4 (*P* < 0.05). Those terms that were also enriched in other samples are shown as green, red, and yellow bars. The lack of a bar indicates that the fraction of upregulated genes in a GO term is not significantly higher than the overall fraction of upregulated genes. The numbers in brackets denote the count of enriched GO terms.

**FIGURE 5:**
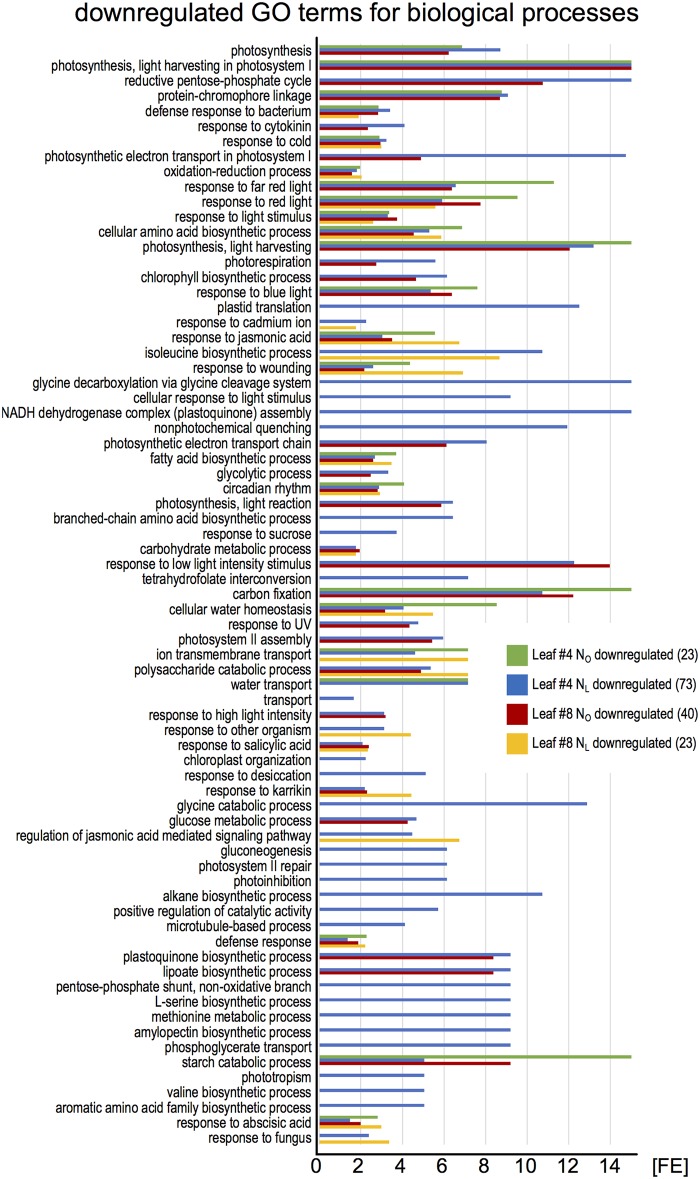
Enriched Gene Ontology terms among downregulated leaf #4 genes. The downregulated genes in all samples (green pictograms in **Figure 2**) were analyzed for enriched Gene Ontology terms using the DAVID Bioinformatics Resources 6.8. The blue bars represent all significantly enriched GO_BP_DIRECT terms in N_L_ leaves #4 (*P* < 0.05). Those terms that were also enriched in other samples are shown as green, red, and yellow bars. The lack of a bar indicates that the fraction of downregulated genes in a GO term is not significantly higher than the overall fraction of downregulated genes. The numbers in brackets denote the count of enriched GO terms.

To identify the most highly regulated N deficiency-responsive and senescence-associated pathways in leaves #4 and #8, we performed a Gene Ontology term enrichment analysis with the up- and downregulated genes in the two leaf #4 STEM clusters and in leaf #8 (**Figure 3**). To visualize considerably regulated biological processes in N-deficient leaves #4, which show the most progressed senescence symptoms, all significantly enriched (*P* < 0.05) GO terms for the up- and downregulated genes are displayed in **Figures 4**, **5**, respectively, and listed in Supplementary Table 9. The most significantly upregulated processes in N_L_ leaves #4 include cell wall weakening, cellular response to N starvation, intracellular bulk degradation of cytoplasmic components like chloroplasts and mitochondria (autophagy, mitophagy) and general leaf senescence activities. N_L_ leaves #4 shared almost 40% of upregulated GO terms with N_O_ leaves #8, but only 19 and 13% with N_O_ leaves #4 and N_L_ leaves #8, respectively. Of the 73 significantly downregulated GO terms in N_L_ leaves #4, approximately one quarter are associated with photosynthesis and related pathways, and another ∼20% encompass biosynthesis of chlorophyll, amino acids, fatty acids, glucose, amylopectin, alkanes, and plastoquinone. In optimally N-supplied leaves #8, 40 of these GO terms (55%) were also downregulated, but only ∼30% in N_O_ leaves #4 and in N-deficient leaves #8. In agreement with photosynthetic gene and senescence marker gene expression levels (**Figure 2** and Supplementary Table 7), the GO term analysis suggests that senescence was most advanced in N_L_ leaves #4 at 99 DAS, followed by N_O_ leaves #8 at 106 DAS, N_L_ leaves #8 at 106 DAS and N_O_ leaves #4 at 99 DAS. Accordingly, N-deficiency induced in developmentally old leaves of the spring OSR cultivar ‘Mozart’ the accelerated progression of senescence and remobilization of nutrients, whereas in developmentally younger leaves in the upper canopy it led to a delay in senescence progression.

In an independent approach to identify up- or downregulated biological pathways, the temporally regulated genes in N_O_ and N_L_ leaves #4 were grouped by their putative biological functions according to the MapMan classification (Thimm et al., 2004), and categories enriched or depleted for regulated genes were identified. Although the total number of regulated genes was five times higher in N_L_ compared to N_O_ plants, the majority of functional gene categories showed no significant differences in the fractions of regulated genes (Supplementary Figure 2). This is consistent with the weak phenotypic differences between N_O_ and N_L_ plants (Supplementary Figure 1). However, major differences are apparent in the categories tetrapyrrole synthesis and photosynthesis, oxidative pentose phosphate pathway (OPP) and C1-metabolism. In leaves #4 of N_L_ plants, half of the genes associated with photosynthesis (97 of 206 genes in this category) and 25% of the genes involved in chlorophyll biosynthesis (12 of 48 genes in the tetrapyrrole category) were downregulated, whereas in N_O_ plants only 5% of the photosynthesis and no chlorophyll biosynthesis genes were downregulated. Thus, the gene expression data reflect the reduced chlorophyll content in N_L_ plants (**Figure 2**). Also downregulated in N_L_, but not in N_O_ plants, were the oxidative pentose phosphate pathway, which generates reductants required for various biosynthetic processes, including fatty acid synthesis and inorganic N and S assimilation (Kruger and von Schaewen, 2003; Bussell et al., 2013) and the one-carbon (C1) metabolism pathway. This pathway is also connected to the S-assimilation pathway by supplying C1 units for the synthesis of *S*-methylmethionine (SMM), which is transported from source leaves via the phloem to sink organs (Hanson and Roje, 2001). The MapMan classification of up- and downregulated leaf #8 genes revealed overall less differences in significantly regulated categories between N_O_ and N_L_ plants compared to the older leaves #4 (Supplementary Figure 3). However, noticeable differences are apparent in the categories tetrapyrrole synthesis and photosynthesis. Opposite to leaves #4, in leaves #8 in both categories a large fraction of the genes was downregulated in N_O_, but not in N_L_ plants, suggesting that, although the chlorophyll content had not yet much declined between 92 DAS and 106 DAS, senescence was more advanced in leaves #8 of plants grown under optimal N supply than in plants grown under reduced N fertilization.

### Senescence- and N Deficiency-Associated Transcription Factor Genes

**FIGURE 6:**
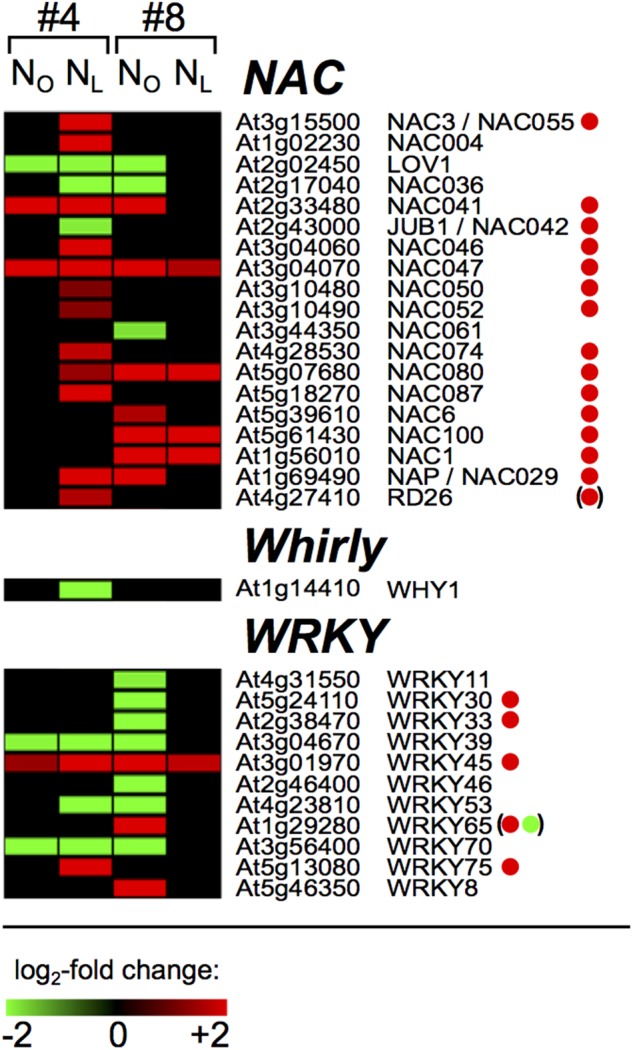
Senescence associated oilseed rape NAC, Whirly and WRKY transcription factor genes. The heat maps display the upregulated (red bars) or downregulated (green bars) *Brassica napus* unigene homologs of *Arabidopsis thaliana* NAC, Whirly and WRKY family transcription factors in leaf #4 between 92 and 99 DAS and in leaf #8 between 92 and 106 DAS as indicated on top of the columns. Depicted are genes with expression ratios ≥ 3 and *P*adj < 0.05 (*n* = 3). Green and red dots denote genes that were reported as leaf senescence-associated down- and upregulated in *Arabidopsis thaliana* by Breeze et al. (2011). Dots in parentheses indicate that this gene was not steadily regulated and *WRKY65* was first up- and later downregulated in the course of leaf senescence.

A major process during leaf senescence is remobilization of N and other nutritional degradation products from source to sink organs. The initiation and progression of senescence is orchestrated by transcription factors (TFs) and thus the identification of senescence-associated TFs that are responsive to N deficiency conditions is crucial for understanding the parameters that determine the N-efficiency of oilseed rape. We therefore investigated the range of *B. napus* homologs of *Arabidopsis* TFs that were differentially regulated upon N-deprivation in source leaves #4 and sink leaves #8. In total, 271 regulated OSR homologs of *Arabidopsis* TFs were found in 37 of the 51 *Arabidopsis* TF families (Yilmaz et al., 2011), and in most families more genes were down- than upregulated (Supplementary Table 10). Under both N-regimes, almost all TF genes in leaf #4 were regulated exclusively in week 3, only nine genes showed regulation during weeks 1 or 2 (Supplementary Table 11). Analogous to the frequencies of total regulated genes (**Figure 1**), between 92 and 99 DAS in N-deficient leaves #4, 3.6-fold more putative TF genes were transcriptionally regulated (78 genes up and 104 genes down) than in optimally N-supplied leaves #4 (22 genes up, 29 genes down). In leaf #8, between 92 and 106 DAS threefold more TF genes were regulated in N_O_ (48 genes up, 92 genes down) than in N_L_ plants (15 genes up, 21 genes down). None of the TF genes were oppositely regulated in N_O_ and N_L_ leaves #4 or leaves #8, or in N_L_ leaf #4 and N_O_ leaf #8. Twelve genes were upregulated exclusively in the senescing N_L_ #4 and N_O_ #8 leaves, among them the *NAP/NAC029* homolog, and thus are leaf rank-independent senescence-associated TFs. Twenty-four TF genes were downregulated solely in the N_L_ #4 and N_O_ #8 leaves, among them the *WRKY53* homolog, suggesting that they are controlling pathways that are downregulated during senescence. The WRKY, Whirly and NAC families deserve special attention, because members of these families were reported to play key roles in controlling leaf senescence and plastid stability in *Arabidopsis*. In contrast to most other TF families, the NAC genes were predominantly upregulated in senescing leaves #4 and #8 (**Figure 6** and Supplementary Table 11). With the only exception of *JUB1*, in senescing *Arabidopsis* leaves the corresponding genes are also upregulated (Breeze et al., 2011). Eleven of the 19 regulated OSR NAC genes were specifically regulated in N_L_ leaves #4 or N_O_ leaves #8, which indicates differences in the regulation of downstream processes in the two canopy levels. Other than the NAC factors, most of the regulated WRKY genes were downregulated and it appears that in this TF family more transcriptional reprogramming occured in N_O_ leaves #8 than in N_L_ leaves #4 (**Figure 6**).

### N-Deficiency Associated Expression of Protein Degradation Genes

The plant-specific developmental process of leaf senescence safeguards the coordinate degradation of proteins, lipids and nucleic acids and remobilization of the resulting low molecular weight nutrients from the senescing leaves to sink organs. Chloroplasts are the most important resource for nitrogen remobilized from senescing source leaves, and autophagy is a crucial process for degradation of chloroplasts during senescence and in response to starvation (Ishida et al., 2014; Michaeli et al., 2014; Izumi et al., 2017). Autophagy mutant plants suffer from premature senescence accompanied by accelerated cell death (reviewed in Minina et al., 2014). Senescence is also accompanied by the activation of various peptidases. To identify the senescence-associated OSR homologs of *Arabidopsis* autophagy genes in leaves #4 and #8 we matched them against the autophagy database (Homma et al., 2011).

**FIGURE 7:**
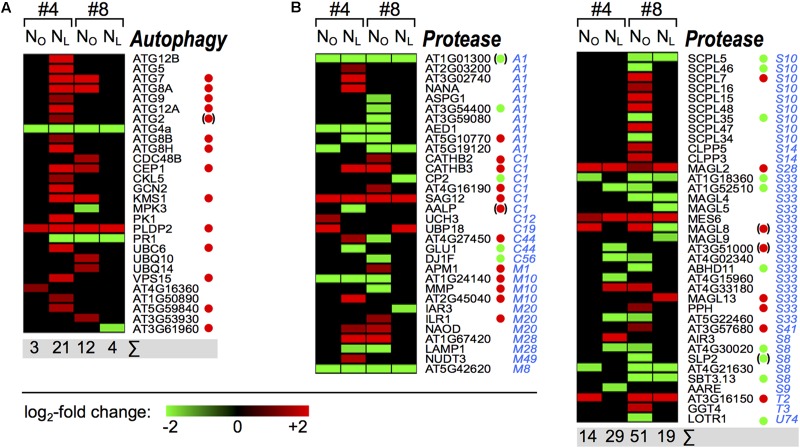
Senescence associated oilseed rape autophagy and protease genes. The heat maps display the upregulated (red bars) or downregulated (green bars) *Brassica napus* unigene homologs of *Arabidopsis thaliana* **(A)** autophagy genes and **(B)** peptidase genes in leaf #4 between 92 and 99 DAS and in leaf #8 between 92 and 106 DAS as indicated on top of the columns. Peptidase family affiliations (MEROPS Peptidase Database) are indicated in blue letters. Depicted are genes with expression ratios ≥ 3 and *P*_adj_ < 0.05 (*n* = 3). Green and red dots denote genes that were reported as leaf senescence-associated down- and upregulated in *Arabidopsis thaliana* by Breeze et al. (2011). Dots in parentheses indicate that this gene was not steadily regulated in the course of leaf senescence.

We identified 28 OSR homologs of *Arabidopsis* autophagy(-related) genes that showed ≥ 3-fold changes in transcription levels (**Figure 7A** and Supplementary Table 12). In N-deficient leaves #4, 19 autophagy gene homologs were upregulated, among them ten ATG core genes that are essential for autophagosome formation (reviewed in Michaeli et al., 2016; Have et al., 2017). Seven of these autophagy core genes and seven autophagy-related genes are also upregulated in senescing *Arabidopsis* leaves (van der Graaff et al., 2006; Breeze et al., 2011). Remarkably, although also in N_O_ leaves #4 senescence initiated during week 3 of the observation period, as was indicated by marker gene expression (**Figure 2**) and enrichment of the GO term ‘leaf senescence’ (**Figure 4**), except for *ATG4a* (see below) none of the autophagy core genes were regulated yet in week 3. Also in N-deficient leaves #8, where senescence was delayed, no activation of the autophagy genes was observed. In N_O_ leaves #8, *ATG7*, *ATG8a* and six autophagy-related genes were upregulated, however, the intensification of the autophagy pathway was clearly lower than in N-deficient leaves #4. Only two genes were regulated in both leaves #4 and #8 and independent of the N supply. *ATG4a*, which encodes a cysteine protease involved in the ATG8 ubiquitination-like pathway and is linked to autophagosome formation, was downregulated in all four samples. In *Arabidopsis*, *ATG4a* is transcriptionally induced by sudden N-depletion and carbon-starvation (Yoshimoto et al., 2004; Rose et al., 2006), but it has not been reported in the context of senescence yet. *PLDP2* was upregulated in all four samples. This gene is also in *Arabidopsis* upregulated during senescence and was reported to regulate vesicle trafficking and to play a role in Pi-starvation (Breeze et al., 2011). The downregulation of the salicylic-acid responsive *PR1* gene (Ward et al., 1991) in all samples except N_O_ leaves #4 is consistent with the corresponding downregulation of the GO term ‘response to salicylic acid’ in these samples.

In addition to autophagy-related proteins, transcriptional and proteomic studies identified in various plant species a large number of senescence-associated, mostly upregulated peptidases from diverse families (reviewed in Roberts et al., 2012). Yet, in winter OSR, only few senescence-associated proteases and protease inhibitors were reported (Etienne et al., 2007; Desclos et al., 2009). We identified in spring OSR ‘Mozart’ overall 69 up- or downregulated OSR homologs of all *A. thaliana* peptidases listed in the MEROPS peptidase database (Rawlings et al., 2016) (**Figure 7B**). For 35 of these, Breeze et al. (2011) observed leaf senescence-associated regulation of the corresponding *Arabidopsis* genes; 30 of them were regulated in the same direction. Other than with the autophagy-related proteins, regulation of protease genes was heterogeneous and more genes were down- than upregulated (37 vs. 32). Remarkably, in contrast to all other gene classes, the highest number of regulated protease genes occured in N_O_ leaves #8. It is tempting to speculate that in spite of the onset of senescence under ample N supply in leaves #8 (**Figure 4**), dismantling of chloroplasts and degradation of chlorophyll is still pending (**Figure 2**) and therefore autophagy is not massively upregulated yet (**Figure 7A**). However, at that stage leaves #8 likely act already as source leaves and provide nutrients for pod development and seed filling (Supplementary Figure 1, Franzaring et al., 2011). The prominent regulation of many proteases in these leaves may be associated with an elevated nutrient export activity.

## Discussion

### Transcriptome Reprogramming in N Supply-Dependent Senescence of Lower and Upper Node *B. napus* Leaves

Since the divergence of the ancestral Brassicaceae into the *Arabidopsis* and *Brassica* lineages ∼17 million years ago (Cheung et al., 2009), genome triplication, allopolyploidization of the *B. napus* parental *B. rapa* and *B. oleracea* genomes, and gene loss events occurred, with the consequence that the modern OSR genome contains zero to more than six orthologs of any *Arabidopsis* gene (Rana et al., 2004). This complicates the identification of orthology relationships between *Arabidopsis* and *B. napus* genes and prevents the distinction between multiple related *B. napus* genes when using *Arabidopsis* microarrays. We therefore used a microarray with 60 nt-probes based predominantly on three EST libraries from *B. napus*, *B. rapa*, and *B. oleracea* and a smaller number of other publically available ESTs (Trick et al., 2009).

In previous studies of the transcriptome response to N starvation in *Arabidopsis thaliana* (Wang et al., 2003; Scheible et al., 2004; Balazadeh et al., 2014) and winter oilseed rape (Koeslin-Findeklee et al., 2015b), plants were grown hydroponically in low N medium and transcription analysis was performed after nitrate re-addition. This treatment may invoke a rapid and temporal plant response to nutrient shock (Wang et al., 2001; Peng et al., 2007) and thus may not fully reflect the plant adaptive responses to long-term low N conditions. Here, we compared the OSR transcriptome in plants of a spring cultivar grown under optimal or low N fertilization in solid medium under seasonal climate simulating conditions, which is more similar to field conditions (Franzaring et al., 2012).

In the developmentally early winter OSR leaves, senescence typically begins during flowering but before the seed filling stage, and the leaves are shed before the developing reproductive organs have reached their maximal sink strength. This is considered as one reason for the relatively inefficient N remobilization and high residual N content in the fallen leaves that, moreover, increases with N fertilization (Schjoerring et al., 1995; Hocking et al., 1997; Rossato et al., 2001; Noquet et al., 2004; Malagoli et al., 2005a). In cauline leaves in the upper canopy, senescence initiates later and the N content of fallen leaves is lower, indicating a more efficient N remobilization from these leaves driven by the higher sink strength of the developing pods during seed filling (Malagoli et al., 2005a; Etienne et al., 2007). In this study we aimed to determine if the chronology of senescence initiation in different canopy levels is similar in a spring OSR cultivar and how differences between early and late leaves are reflected in their transcriptomes.

Taking the expression changes of the senescence marker genes *BnSAG12-1*, *BnSAG2* and *BnCAB1* as indicators, under optimal as well as low N supply the first signs of senescence initiation appeared in the lower canopy leaf #4 already in week 1 of the observation period. In this early senescence phase the chlorophyll content is not a useful indicator for senescence or N deprivation (**Figure 2**), as had also been observed by Gombert et al. (2006). In the following 2 weeks senescence progressed under both N regimes, but more rapidly in the N deficient plants as is indicated by the massive downregulation of PSGs. In the upper canopy leaf #8 the *BnSAG12-1*, *BnSAG2* and *BnD22* expression changes did not show a difference in the senescence status of N_O_ and N_L_ leaves. However, downregulation of *BnCAB* and many PSGs indicated that N deficiency led to a delay of senescence progression in younger leaves. This conclusion was corroborated by the extent of transcriptome reprogramming and the affected metabolic processes. In leaves #4 essentially no change in gene regulation in either N_O_ or N_L_ leaves was observed in week 1. One week later at an overall low level already twice as many genes were regulated in N_L_ compared to N_O_ leaves, and in week 3 in N-deficient leaves the number of regulated genes increased another 15-times to almost 3,000 regulated genes, whereas under ample N supply less than 600 genes were regulated. The effect of N deprivation was opposite in the upper canopy leaves #8, where 2.5-times more genes were regulated in N_O_ plants. The functional classification of regulated genes revealed that senescence-associated transcriptome reprogramming in spring oilseed rape cv. ‘Mozart’ comprises largely the same biological processes as in *Arabidopsis thaliana* (Buchanan-Wollaston et al., 2005; van der Graaff et al., 2006; Breeze et al., 2011).

### Divergent Regulation of Transcription Factors in Senescing Young and Old Leaves

The age-dependent expression of thousands of senescence-associated genes is orchestrated by transcription factors, many of which are themselves transcriptionally regulated during senescence. Several of these TFs are also induced by various biotic or abiotic stresses, indicating that senescence is an integrated response of plants to endogenous developmental signals and environmental cues (Woo et al., 2013). In *Arabidopsis*, transcriptomic analyses revealed the enrichment of upregulated TF genes of the NAC, WRKY, AP2/EREBP, MYB, C2H2 zinc-finger, bZIP, and GRAS families during leaf senescence (Buchanan-Wollaston et al., 2005; van der Graaff et al., 2006; Balazadeh et al., 2008, 2010; Breeze et al., 2011). We observed also in spring OSR a senescence-associated transcriptional reorganization in all these TF families. The comparison of the expression changes of the 112 senescence-associated TF genes that were identified in *Arabidopsis* by Breeze et al. (2011) and here in OSR reveals largely congruent transcription increases or decreases (Supplementary Table 11). Interestingly though, we note in OSR that 107 TF genes were only in N_L_ leaf #4 and 65 genes only in N_O_ leaves #8 more than threefold up- or downregulated, which indicates distinct regulation of individual senescence processes and nutrient remobilization in upper and lower canopy leaves. Also noticeable is a virtually perfect congruence of TF gene regulation in certain families between OSR and *Arabidopsis* (NAC, C2C2, C2H2) and more divergent regulation in others (AP2-EREBP, C3H, homeobox).

The expression profiles of well characterized key regulators of senescence in OSR and *Arabidopsis* attest that, in spite of their very different sporophyte architectures, the regulatory network controlling senescence is similar in these two Brassicaceae. For example, in both species the positive regulators of chlorophyll degradation *NAC046* and *NAC055* and the senescence promoting *NAP/NAC029* factor are upregulated (**Figure 6**) (Guo and Gan, 2006; Hickman et al., 2013; Oda-Yamamizo et al., 2016). On the other hand, the negative regulator of senescence *WRKY70* (Ülker et al., 2007; Zentgraf et al., 2010; Besseau et al., 2012) and the early induced *WRKY53* factor, which interacts with other senescence regulators (Hinderhofer and Zentgraf, 2001; Miao et al., 2004; Zentgraf et al., 2010), were downregulated. However, in a few cases also divergent regulation of senescence-controlling factors in *Arabidopsis* and OSR was observed. The NAC family member *JUB1*, which was identified as a longevity-promoting factor in *Arabidopsis* (Wu et al., 2012), was downregulated in N_L_ leaves #4. Surprisingly, it was found to be induced in *Arabidopsis* during leaf senescence (Breeze et al., 2011). Noteworthy is also the downregulation of the OSR homolog of *WHY1* in N_L_ leaves #4 (**Figure 6**), because this gene is involved in maintaining chloroplast stability. The *Arabidopsis WHY1* gene, which is one of only three Whirly family genes in this plant, is required for chloroplast genome stability (Marechal et al., 2009), and the barley *WHIRLY1* ortholog is involved in premature senescence induction under photooxidative stress (Kucharewicz et al., 2017).

### Chloroplast Decomposition and Protein Degradation Pathway Activation in Senescing Leaves

The critical role of autophagy for the disassembly of chloroplasts, mitochondria and other cellular structures in the course of senescence has been extensively demonstrated in *Arabidopsis* (reviewed by Michaeli et al., 2016; Have et al., 2017). During developmental and starvation-induced senescence, entire chloroplasts can be degraded by autophagy (Minamikawa et al., 2001; Wada et al., 2009). Other than in *A. thaliana*, where 9 of 15 upregulated autophagy genes were activated in leaves that were not even fully expanded yet and showed no signs of senescence (Breeze et al., 2011), in OSR we do not observe activation of autophagy genes in N_O_ leaves #4 or N_L_ leaves #8, while senescence was initiated in these leaves. However, in the more advanced senescence stages in N_L_ #4 and N_O_ #8 leaves, more OSR autophagy genes appear to be upregulated than in *Arabidopsis* (Breeze et al., 2011), although we did not consider all statistically significantly regulated genes but only those exhibiting ≥ 3-fold transcriptional changes. A possible explanation could be that autophagy is a generic, auto-cleaning process required to remove obsolete cell components and maintain cellular integrity. It is thus constitutively active at a low level which might be sufficient during early senescence. Only when senescence progresses it may become necessary to boost the autophagy pathway.

The more pronounced transcriptional activation of autophagy genes in N-deficient leaves #4 compared to N_O_ leaves #8 could indicate that the cell death program, the last phase of senescence, has started in the lower canopy leaves, whereas the young upper canopy leaves #8 have to stay alive to serve as source leaves for nutrient remobilization toward the developing pods. This course of events is known from winter OSR genotypes (reviewed in Avice and Etienne, 2014). Consistent with this hypothesis is the much higher number of regulated protease genes in N_O_ leaves #8 which may be involved in protein turnover and N remobilization, but not in executing cell death.

Differences between OSR and *Arabidopsis* are also apparent in the regulation of senescence-associated peptidase genes, which play a crucial role in providing nitrogen transport molecules like amino acids for developing sink organs (reviewed by Masclaux-Daubresse et al., 2010). Similar to the group of autophagy genes, in OSR more peptidase genes are differentially regulated than in *Arabidopsis* (Breeze et al., 2011), and a larger fraction of these genes is downregulated. These differences might indicate a partly different orchestration of the senescence course in OSR, which may reflect the more complex architecture and morphological development of OSR plants compared to *A. thaliana*. Recently, by protease activity profiling Poret et al. (2016) identified in senescing *B. napus* leaves after 23 days of N-starvation an activity increase relative to plants grown with ample N-supply of 17 serine- and cysteine-proteases with homology to 10 *Arabidopsis* proteases including SAG12, AALP, and AARE. In our study, both AALP and AARE are downregulated only in N-deficient leaves #4 (**Figure 7B**). However, transcription data do not always reflect protein level or activity data, as has also been reported for metabolic flux data (Schwender et al., 2014), and especially proteases are frequently regulated at the post-transcriptional level.

## Conclusion

We found evidence that the sequence of senescence initiation and progression and also the effects of N-limitation are similar in the spring OSR cultivar ‘Mozart’ and in winter OSR cultivars. Like in winter OSR, long-term, mild N deficiency leads in spring OSR to premature shutdown of PSGs and senescence in lower canopy source leaves, whereas in upper canopy sink leaves senescence progression is delayed. The onset of senescence is accompanied by a massive reprogramming of the transcriptome. The affected regulatory and metabolic pathways are overall similar to those in *Arabidopsis*, but we identified transcription regulator and protein degradation genes that are specifically regulated in N-depleted lower canopy leaves or in upper leaves under ample N supply, and genes that are senescence-associatedly expressed in oilseed rape, but not in *Arabidopsis*. In future studies it will be interesting to address the question whether these genes fulfill specific tasks in N-remobilization during N deficiency-induced leaf senescence and if their regulation affects the nitrogen use efficiency of oilseed rape.

## Author Contributions

VS-R: acquisition, analysis, and interpretation of data; writing the manuscript. JF: acquisition of data and design of the work. AF: design of the work. RK: conception and design of the work; acquisition, analysis, and interpretation of data; writing the manuscript.

## Conflict of Interest Statement

The authors declare that the research was conducted in the absence of any commercial or financial relationships that could be construed as a potential conflict of interest.

## Footnotes

1 http://faostat3.fao.org

2 https://www.gesetze-im-internet.de/d_v_2017/

3 http://ec.europa.eu/environment/water/water-nitrates/index_en.html

4 www.sourcebioscience.com

5 www.mbio.ncsu.edu/BioEdit/bioedit.html

6 www.bioconductor.org/packages/release/bioc/html/limma.html

7 http://bar.utoronto.ca/ntools/cgi-bin/ntools_classification_superviewer.cgi

8 https://david.ncifcrf.gov

9 www.tm4.org/

10 http://arabidopsis.med.ohio-state.edu

11 www.tanpaku.org/autophagy/index.html

12 http://merops.sanger.ac.uk

13 http://quantprime.mpimp-golm.mpg.de
