# Supplementary material for: Divergent N Deficiency-Dependent Senescence and Transcriptome Response in Developmentally Old and Young Brassica napus Leaves
Source: Front Plant Sci. 2018 Feb 1;9:48. doi: 10.3389/fpls.2018.00048 (PMC5799827; doi:10.3389/fpls.2018.00048)
Supplement: Supplementary file 2 [file Data_Sheet_1.PDF]

*Supplementary Material*

# Divergent N deficiency-dependent senescence and transcriptome response in developmentally old and young *Brassica napus* leaves

**Vajiheh Safavi-Rizi, Jürgen Franzaring, Andreas Fangmeier and Reinhard Kunze***

*** Correspondence:** Reinhard Kunze: reinhard.kunze@fu-berlin.de

## Supplementary Figures

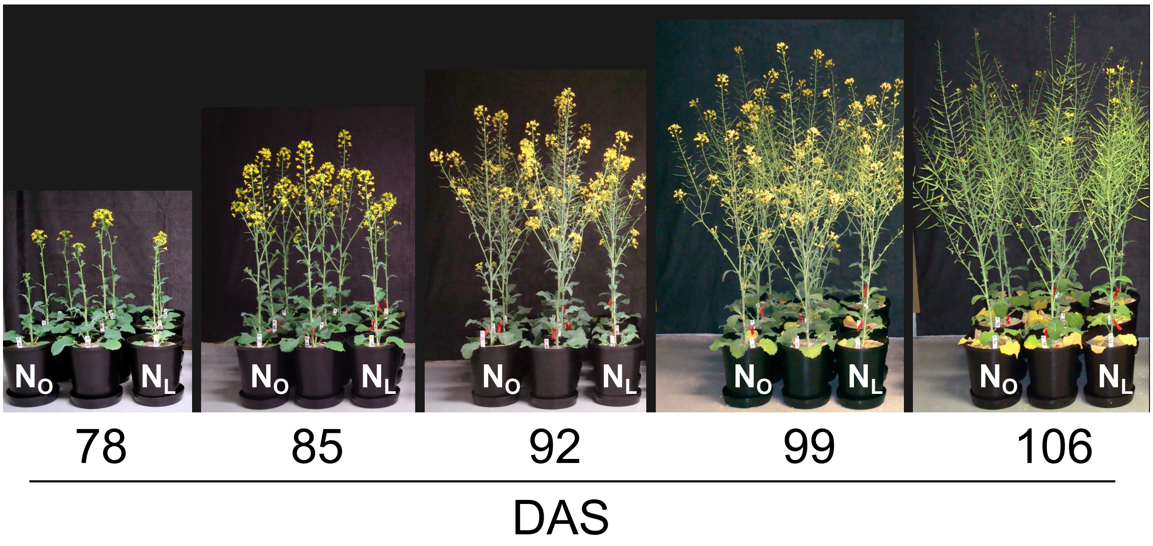


**Supplementary Figure 1. Phenotype of *B. napus* cv. Mozart plants grown under optimal or low N supply.** In each panel the three plants on the left were grown under optimal N supply ($N_O$) and the three plants on the right were grown under low N supply ($N_L$). The three plants in the center (grown under elevated N supply) were not used in this study.

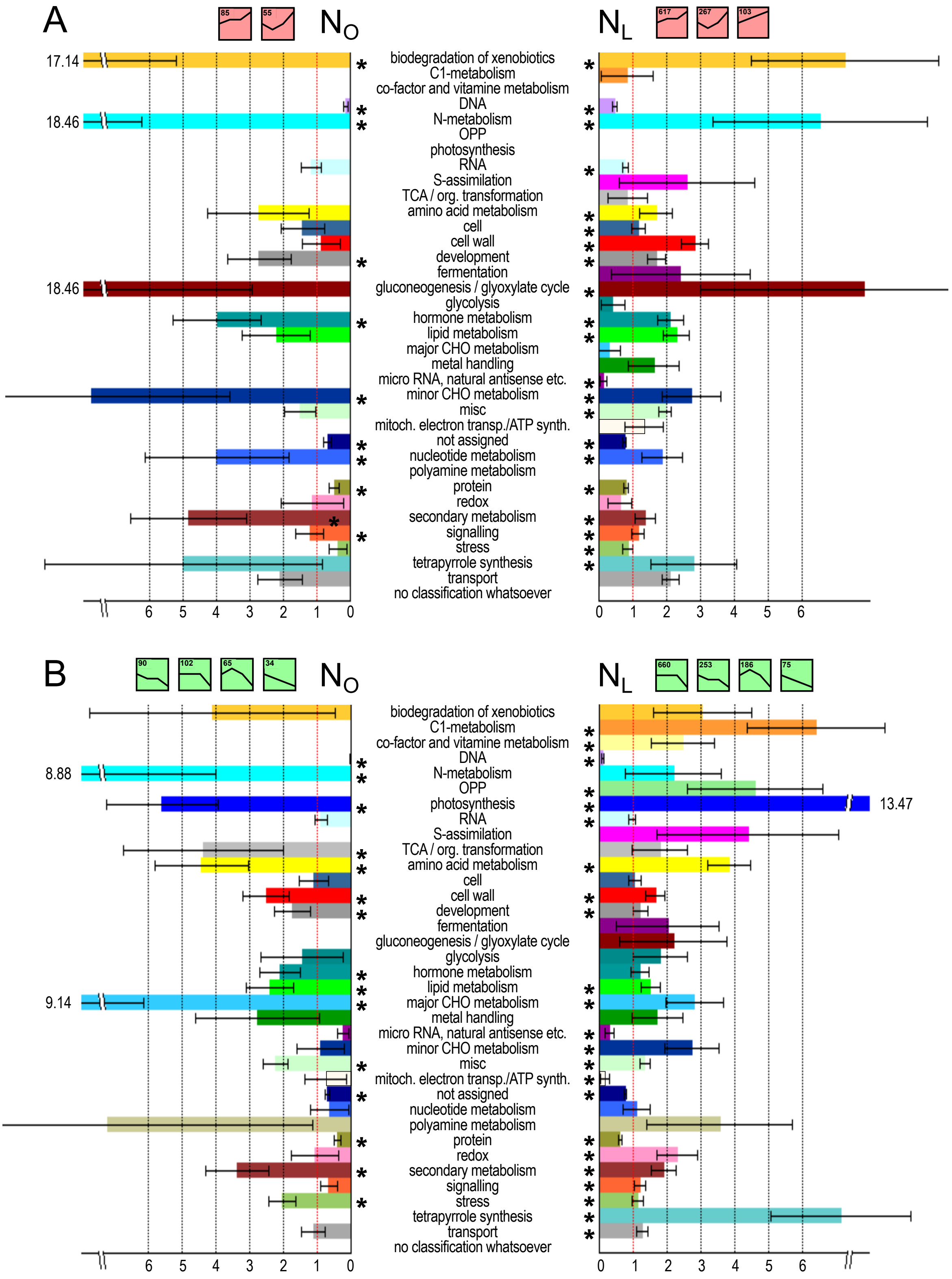


**Supplementary Figure 2. MapMan functional classification of temporally up- and downregulated genes in leaf #4.** Genes in the (**A**) 'downregulated' and (**B**) 'upregulated' STEM cluster of $N_O$ and $N_L$ leaves #4 were classified according to their putative assignment to MapMan metabolic pathways using the BAR Classification SuperViewer Tool. Bars show the normed frequency (NF) of genes in functional categories ± SD for 100 bootstraps. Normed frequency is the (Number in $\text{Class}_{\text{input\_set}}$/Number $\text{Classified}_{\text{input\_set}}$)/(Number in $\text{Class}_{\text{reference\_set}}$/Number $\text{Classified}_{\text{reference\_set}}$). Enriched (NF > 1) or underrepresented categories (NF < 1) which are statistically significant (p-value of the hypergeometric distribution < 0.05) are marked by asterisks.

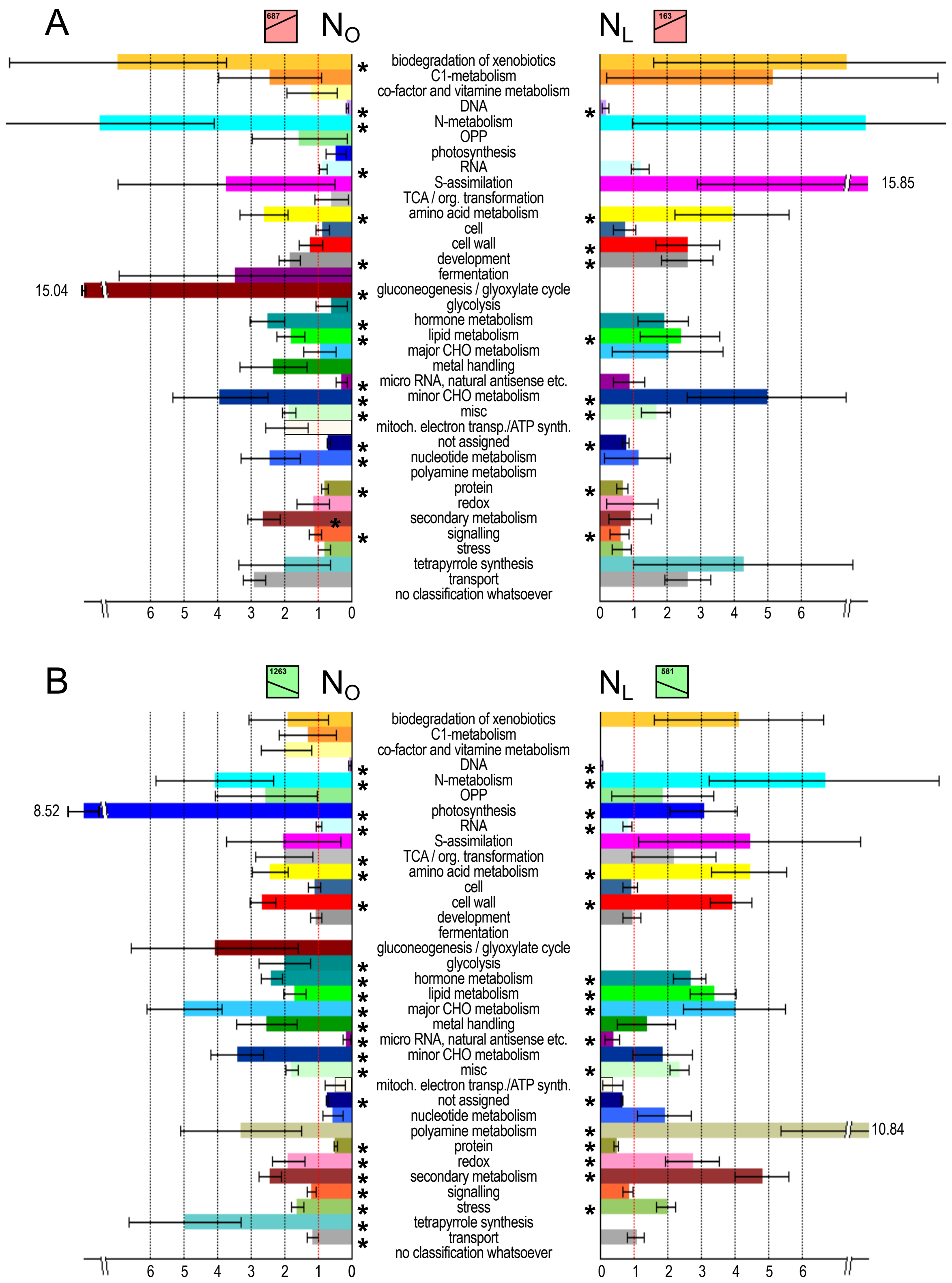


**Supplementary Figure 3. MapMan functional classification of up- and downregulated genes in leaf #8.** Genes in the (**A**) 'downregulated' and (**B**) 'upregulated' $N_O$ and $N_L$ leaves #8 were classified according to their putative assignment to MapMan metabolic pathways using the BAR Classification SuperViewer Tool. Bars show the normed frequency (NF) of genes in functional categories ± SD for 100 bootstraps. Normed frequency is the (Number in $Class_{input\_set}$/Number $Classified_{input\_set}$)/(Number in $Class_{reference\_set}$/Number $Classified_{reference\_set}$). Enriched (NF > 1) or underrepresented categories (NF < 1) which are statistically significant (p-value of the hypergeometric distribution < 0.05) are marked by asterisks.
